# Health Care Seeking Behavior in Southwest Ethiopia

**DOI:** 10.1371/journal.pone.0161014

**Published:** 2016-09-14

**Authors:** Bayu Begashaw, Fasil Tessema, Hailay Abrha Gesesew

**Affiliations:** 1 Department of Public Health, Mizan Tepi University, Mizan Teferi, SNNPR, Ethiopia; 2 Department of Epidemiology, Jimma University, Jimma, Oromiya, Ethiopia; 3 Discipline of Public Health, Faculty of Medicine, Nursing and Health Sciences, Flinders University, Adelaide, South Australia, Australia; National Institute of Health, ITALY

## Abstract

**Background:**

Rural and urban populations have disparate socio-demographic and economic characteristics, which have an influence on equity and their health seeking behavior. We examined and compared the health care seeking behavior for perceived morbidity between urban and rural households in Southwest Ethiopia.

**Methods:**

Analytic cross-sectional study was conducted among urban and rural households living in Esera district of Southwest Ethiopia. A random sample of 388 head of households (126 urban and 262 rural) were selected. A pretested and structured questionnaire was used for data collection with face-to-face interview. In addition to descriptive methods, binary logistic regression was used to identify factors associated with health seeking behavior at p value of less than 0.05.

**Results:**

Of the sample household heads, 377 (97.2%) (119 urban and 258 rural) were successfully interviewed. Among these, 58.4% (95% CI, 53.3–63.3%) of the households sought care from modern health care that was lower among rural (48.1%) than urban (80.7%) households. The prevalence of self-treatment was 35.3% in urban and 46.1% in rural households. Among the factors considered for modern health care utilization, higher monthly income (AOR, 5.6; 95% CI, 2.04–15.4), perceived severity of disease (AOR, 2.5; 95% CI, 1.1–5.8), acute duration of disease (AOR, 8.9; 95% CI, 2.4–33.3) and short distance from health facilities (AOR, 3; 95% CI, 1.2–8.4) among rural and being married (AOR, 11.3; 95% CI, 1.2–110.2) and perceived severity of disease (AOR, 6.6; 95% CI, 1.1–10.9) among urban households showed statistically significant association.

**Conclusions:**

The general health seeking behavior of households on perceived morbidity was satisfactory but lower in rural compared to urban households. Self-medication was also widely practiced in the study area. The findings signal the need to work more on accessibility and promotion of healthcare seeking behavior especially among rural households.

## Background

Health care policies and programs’ planning requires knowledge about health care seeking behavior for early diagnosis, effective treatment and appropriate intervention implementation [1]. Early health care utilization and adherence to effective treatment can reduce morbidity, disability and mortality [2, 3]. However, we have learnt the growing of evidences in inequity and inequality to access to health care services [4]. Previous studies showed that inequality in health care utilization and health outcomes between the poor versus rich and urban versus rural are unjust and unfair [5]. This does not reflect the prohibition or more deserving for poor than rich or rural than urban, rather inequity and inequality should consider variety strengths, challenges, opportunities and threats [6–9]. The problem of partiality in health care utilization is getting sever in Sub-Saharan Africa including Ethiopia[10].

Ethiopia is currently experiencing demographic and epidemiological [11], and nutritional transition[12], which presents a hurdle for designing the health care policies and programs. Thus, nowadays, chronic illnesses are not disease of affluent people or country nor infectious diseases are confined to poor people or countries [1, 11]. There is also a shift from high prevalence of under nutrition to diet related chronic non-communicable diseases (NCDs) [1, 13]. Despite both infectious and non-infectious diseases are becoming common in both urban and rural households, the health care seeking behavior, utilization, and accessibility and availability is disproportionate [14, 15]. The differences in socio-demographic and -economic characteristics between urban and rural areas contribute to the difference in healthcare-seeking behavior [6, 16].

Healthcare-seeking behavior is influenced by availability, quality and price of services as well as to social group, health views, residences and personal features of the users [14, 15]. Besides peoples’ choice of health care differs in sociodemographic, socio-economic and cultural compositions which have an effect on their health care seeking behavior[17]. Urban dwellers are generally believed to be open to new ideas and willing to try certain things on a trial and error basis[18]. To the contrary, rural dwellers are seen as prone to tradition, unchanging and unwelcoming to change, and willing to hang onto traditional values and practices [19–21]. Results from different continents of Europe, Asia and sub Saharan Africa showed that percentage of healthcare seeking behaviour for perceived morbidity in rural setup are still low. For example, in countries like Mongolia [22] and Republic Congo [23], it was reported as 44.1% and 54.6% respectively. In Ethiopia, the prevalence of rural health seeking behavior was reported as 38.7% [19].

Healthcare-seeking behavior is a complex outcome due to the aforementioned factors and needs contextual exploration between urban and rural households so as to frame commendations that will help with the design of health care policies and programmes [1]. Therefore, in this study we compared the proportion and evaluate factors associated with healthcare seeking behaviors between urban and rural households on perceived morbidity.

## Materials and Methods

### Study design, setting and participants

A community based analytic cross–sectional study was carried out between February and March, 2015 in Esera woreda (district), Southwest Ethiopia. Esera woreda is located about 670 km from Addis Ababa, the capital city of Ethiopia. In 2015, the district had an estimated total population of 99,319 with 1:2 urban to rural proportion [24, 25]. It has four urban and 25 rural kebeles (the lowest administrative structure in Ethiopia) with an average of 4.8 persons per household (3.8 in urban and 5.0 in rural households) [24]. There were four health centers, 29 health posts and 117 health professionals in the district.

### Sampling

The required sample size was calculated using double population proportion calculation formula with the following assumptions: 52.3% prevalence of healthcare seeking for perceived illnesses of urban households and 29.6% prevalence of healthcare seeking for perceived illnesses of rural households [26], 95% confidence level, 5% margin of error, 80% power, 2:1 rural to urban ratio and 10% estimated non–response rate. Considering a design effect of 2, the calculated sample size of households was 388 (126 urban and 258 rural).

**Fig 1:**
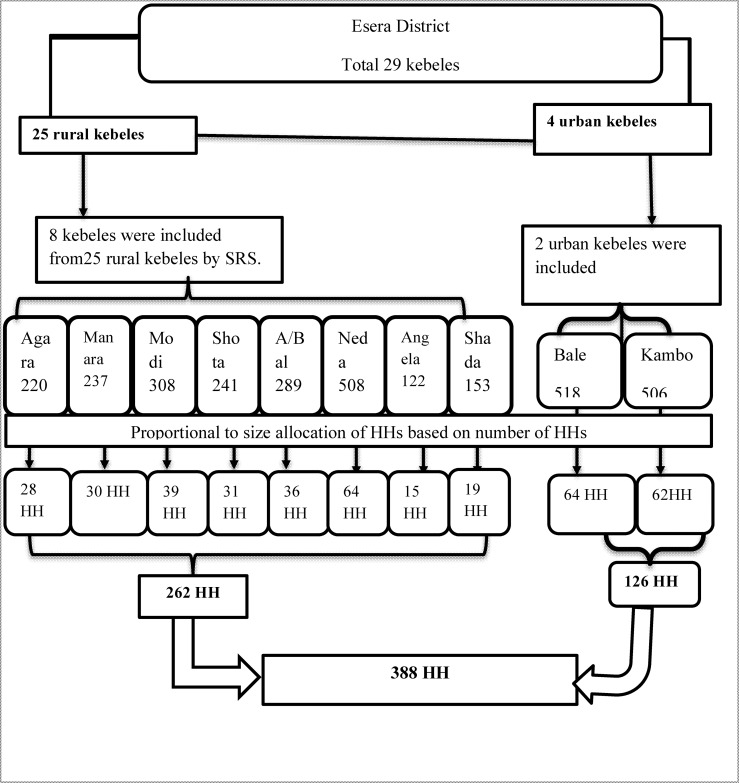
Diagrammatic presentation of sampling technique and procedure. Fig 1 shows the graphic presentation of recruitment of households included in the study. District stratified in to rural and urban; kebeles in both urban and rural selected via SRS; census was conducted among the selected kebeles; and households were allocated via PPS.

Multistage sampling technique was used to recruit the respondents. The district was classified into two strata; urban and rural. Then two urban and eight rural kebeles were randomly selected. To identify households with perceived illness during the last two months, census of households was conducted in the selected kebeles and used as a sampling frame. Then using the sampling frame, households were selected via simple random sampling technique and head of households were interviewed at their home. The schematic presentation of the sampling technique is presented in Fig 1.

### Variables in the study and its measurement

The dependent variable was healthcare seeking behavior coded as 1 if heads claimed sough healthcare in any modern health facility—hospital, health center and private clinic) or 0 otherwise when any member of the household was sick. The exposure variables included age, sex, education, occupation, marital status, family income, distance from health facility, cost of healthcare service, duration of illness, perceived severity of illness, use of traditional medicine and self-medication.

Level of education was classified as illiterate (couldn’t read and write), literate (could read and write but received no formal education), primary (received education up to grade eight), secondary (received education 9–12 grade), and college or university. Duration of illness was measured as acute-if lasted for less than 14 days or chronic- if lasted for more than 14 days. Perceived severity of disease was measured using the question, “Did you think the illness was serious?” and self-medication using the question, “Would you prefer a self-treatment?” with “yes” or “no” responses in both.

Data were collected using face-to-face interview using a structured questionnaire (S1 File) with the head of the households at home level. Data collectors got training on the aim, confidentiality of information, respondent’s right and procedures of interview prior to census and actual study data collection.

### Data analysis

Data exploration, editing and cleaning were undertaken before analysis. The analysis of both descriptive and inferential statistics was conducted. Descriptive statistics included mean and standard deviation values for continuous data; percentage and frequency tables for categorical data. Logistic regression was used to identify factors associated with health seeking behavior. Bivariate logistic regression analysis was conducted to see the existence of crude association and select candidate variables (with P value below 0.25 were considered) to multivariable logistic regression. We checked multi-collinearity among selected independent variables via variance inflation factor (VIF) and none was found. P-value ≤0.05 was considered as a cut point for statistical significance in the final model. Fitness of goodness of the final model was checked by Hosmer and Lemeshow test and was found fit. Three models: urban, rural and both were developed to compare the health seeking behavior of the households. Data were summarized using odds ratio (OR) and 95% confidence interval. Data analysis was conducted using SPSS version 14 for windows.

### Ethical statement

The study was approved by institutional review board of college of health sciences at Jimma University (Approval number: RPGC/558/2015). Permission for the study to be conducted was also obtained from the kebeles. Participants were informed of the study and its purpose in their mother tongue. Study participants gave an informed consent before the commencement of each interview, and no personal identification was registered. We prepared an informed verbal consent that involved purpose of the research, expected duration of the interview, and a description that the participants could withdraw from the interview at any time, had no risk and no payment for their recruitment. This statement was read to each study participants before conducting the interview and requested their permission to be involved in the study.

Verbal consent was proposed over written consent for the following reasons. Firstly, this was cross-sectional study that enquired descriptive data. Secondly, their responses had no personal, social or political consequences. Thirdly, there would not be significant risk/s to the participants. Lastly, a significant number of people living in the rural areas in Ethiopia have no educational status. The IRB approved the proposed verbal consent procedure. The Confidentiality of the data was ensured.

## Results

### Characteristics of study participants

**Fig 2:**
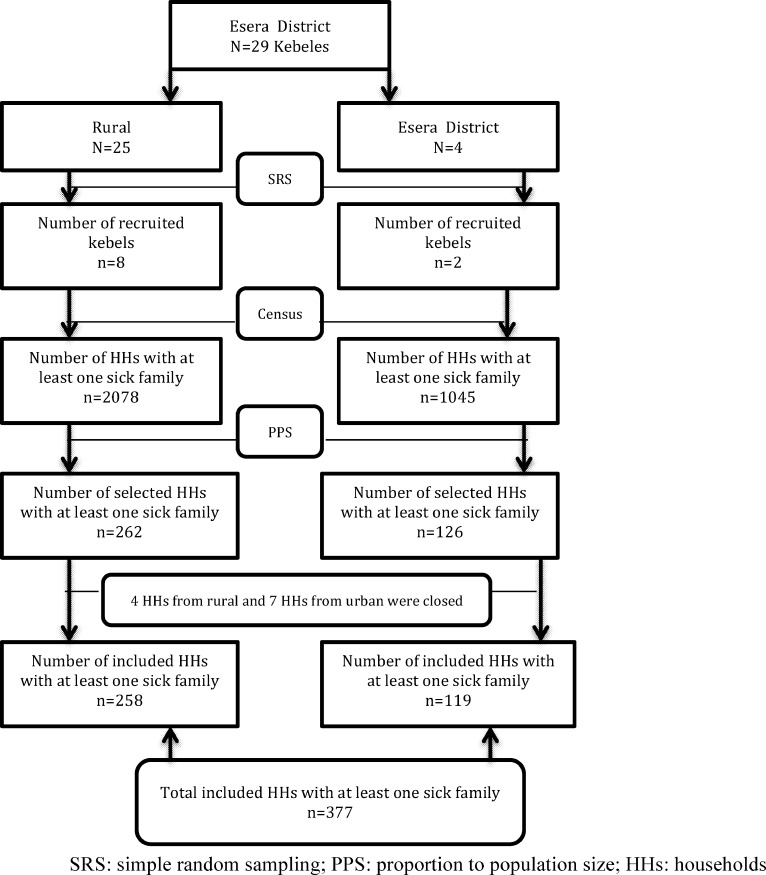
Graphic presentation of HHs (household) recruitment, Esera district, Southwest Ethiopia, 2014. Fig 2 shows the graphic presentation of recruitment of households included in the study. District stratified in to rural and urban; kebeles in both urban and rural selected via SRS; census was conducted among the selected kebeles; and households were allocated via PPS. Finally, number of HHS included in the sample was described and reason of non-respondents was included.

Following the census, 3123 households (1045 urban and 2078 rural) were found to have at least one sick family member within the last two months prior to the study of which 388 were selected randomly. Of the sampled households, 377 (97.2%) participants- 119 urban heads and 258 rural heads- participated in the interview (Fig 2).

Table 1 shows demographic and economic characteristics of the respondents. Similar age distribution was observed in urban (mean 37.5 (SD = 12.9) years) and rural households (mean 36.1(SD = 12.4) years). Majority of the respondents were males in both urban (72.3%) and rural (67.1%) households and married 94.4% in urban and 66.7% in rural households. Nearly half of urban households were employed in private work whereas 80% rural households were farmers. Only 40(33.6%) urban and 13(5%) rural household heads attended college education and above. Two third (68.6%) of rural and one third (38.7%) of urban households had family size greater than four. Eighteen (18.4%) urban and 170(65.8%) rural households had a monthly income below 1170 Ethiopian Birr (ETB).

**Table 1:** Demographic characteristics of urban and rural households in Esera district, Southwest Ethiopia, 2015.

Characteristics (n = 377)		Urban, n (%)	Rural, n (%)	Total, n (%)
**Age in years**	18–30	61(51.3)	100(38.8)	161(42.7)
	31–45	47(39.5)	90(34.9)	137(36.3)
	46–59	7(5.9)	50(19.4)	57(15.2)
	60+	4(3.3)	18(6.9)	22(5.8)
	Mean (years)	33 (±10.7)	37 (±12.8)	36 (±12.4)
**Sex**	Male	86(72.3)	173(67.1)	259(68.7)
	Female	33(27.7)	85(32.9)	118(31.3)
**Matrimonial status**	Single	28(23.5)	39(15.1)	67(17.8)
	Married	72(60.5)	186(72.1)	258(68.4)
	Widowed	8(6.7)	17(6.6)	25(6.6)
	Divorced	11(9.3)	16(6.2)	27(7.2)
**Marital structure (n = 258)**	Monogamy	68(94.4)	124(66.7)	192(74.5)
	Polygamy	4(5.6)	62(33.7)	66(25.5)
**Religion**	Orthodox	57(47.9)	91(35.3)	148(39.3)
	Protestant	51(42.9)	108(41.9)	159(42.2)
	Catholic	5(4.2)	38(14.7)	43(11.4)
	Muslim	1(0.8)	8(3.1)	9(2.4)
	Others	5(4.2)	13(5)	18(4.7)
**Occupation**^**a**^	Farmer	16(13.4)	212(82.2)	228(60.5)
	Government employee	39(32.8)	13(5)	102(27.1)
	Private employee	58(48.7)	21(8.1)	144(38.2)
	Other^b^	6(5.1)	12(4.7)	18(4.8)
**Educational status**	Illiterate	10(8.4)	113(43.8)	123 (32.6)
	Primary	33(27.7)	111(43)	144 (38.2)
	Secondary	36(30.3)	21(8.2)	57 (15.1)
	College and above	40(33.6)	13(5)	53 (14.1)
**Family size**	< 4	73(61.3)	81(31.4)	154(40.8)
	> = 4	46(38.7)	177(68.6)	223(59.2)
**Monthly income (in Birr)**	< 1,170> = 1,170	22(18.5)97(81.5)	169(65.5)89(34.5)	191(50.7)186 (49.3)
**Source of money for health care during illness**	Cash	103(86.5)	46(17.8)	149(39.5)
	Selling agricultural products	15(12.7)	204(79.1)	219(58.1)
	Free care	1(0.8)	8(3.1)	9(2.4)

^a^More than one type of occupation was reported per respondent.

^b^Johvah witness, traditional faith, pagan

### Health care seeking behaviors

Table 2 shows healthcare seeking behaviours of urban and rural households. Accordingly, 105 (88.2%) urban and 218 (84.5%) rural households reported about perceived morbidity, two third of urban and half of rural households perceived acute illness. The general prevalence of health care seeking behavior was 58.4% (95% CI, 53.3–63.3%) with 81% (95% CI, 72.4–87.3%) urban and 49.1% (95% CI, 42.5–55.7%) rural households. Majority of the households (45.3% of urban and 50.5% of rural) sought health care from public health center.

**Table 2:** Health care-seeking behaviors of urban and rural households, Esera district, Southwest Ethiopia, 2015.

Variable		Urban, n (%)	Rural, n (%)	Total, n (%)
**Perceived morbidity**	Yes	105 (88.2)	218 (84.5)	323 (85.6)
	No	14 (11.8)	40 (15.5)	54 (14.4)
**Disease condition**	Acute	67 (63.8)	109 (50)	176 (54.5)
	Chronic	38 (36.2)	109 (50)	147 (45.5)
**Perceived severity**	Yes	78 (74.3)	85 (39.2)	163 (50.6)
	No	27 (25.7)	132 (60.8)	159 (49.4)
**Health care seeking behavior**	Yes	85 (81)	107 (49.1)	192 (59.4)
	No	20 (19)	111 (50.9)	131 (40.6)
**Facility where care sought**	Health post	0	21 (20.6)	21 (10.9)
	Health center	39 (45.3)	54 (50.5)	93 (48.4)
	Hospital	22 (25.6)	7 (6.5)	29 (15.1)
	Private clinics	18 (20.9)	13 (12.1)	31 (16.2)
	Traditional healers	7 (8.1)	11 (10.3)	18 (9.4)
**Time of health seeking**	Immediately as illness started	46 (54.1)	48 (44.9)	94 (48.9)
	When it goes worse	39 (45.9)	59 (55.1)	98 (51.1)
**Self-treatment practice**	Yes	42 (35.3)	119 (46.1)	161 (42.7)
	No	77 (64.7)	139 (53.9)	216 (57.3)
**Outcome of self-treatment**	Successful	25 (59.5)	49 (41.2)	74 (46.0)
	Not successful	17 (40.5)	70 (58.8)	87 (54.0)

Eighteen households visited traditional healing places of which 10 persons were from urban and 20 persons were from rural that included herbalist (14), *‘Tsebel’* (Holy Water) (12), ‘*Wegesha*’ (traditional physiotherapy) (10) and ‘*Kalicha’* (Traditional Psychotherapy) (6) as the commonest places visited by both urban and rural households. Self-treatment was practiced by 42 (35.3%) urban and 119 (46.1%) rural households with the most common reported reason being cost (31.0% urban and 47.5% rural) followed by perceived knowledge of treatment (23.8% urban and 20.0% rural). Of those who practiced self-treatment, more than half (54.0%) were unsuccessful in terms curing, 40.5% for urban and 58.8% for rural households.

### Factors associated with health care seeking behaviors of urban and rural households

Table 3 presents the different models fitted to assess health care seeking behavior. The first model was fitted to assess factors for health seeking behavior of urban households. Accordingly, matrimonial status and perceived severity were independently associated with health seeking behavior in urban households. The odds of health seeking behavior among married participants was 11 times higher than single ones (AOR = 11.3, 95% CI: 1.2, 110.2). Perceiving serious illness was about seven times more likely to seek health care than not perceiving serious illness (AOR = 6.6, 95% CI: 1.1, 10.9).

**Table 3:** Factors associated with health care seeking behaviors of urban, rural, and urban and rural households in Esera woreda, Southwest Ethiopia, 2015.

Variables		Urban (OR, 95% CI)		Rural (OR,95%CI)		Urban and rural (OR, 95%CI)	
		COR	AOR	COR	AOR	COR	AOR
**Educational status**	Illiterate	1	1	1	1	**1**	1
	Primary	1.5(0.3, 2.7)	0.1(0.00, 5.01)	**1.8(1.02, 3.2)**	1.5(0.6, 3.9)	1.6(0.9, 2.8)	1.1(0.5, 2.4)
	Secondary	2.6(0.2, 1.2)	0.8(0.01, 46.2)	**6.4(1.7, 23.9)**	1.4(0.2, 11.1)	**10.5(4.1, 26.8)**	2.6(0.7, 9.6)
	College and above	4.4(0.3, 9.7)	1.2(0.02, 96.7)	3.7(0.9, 15.2)	0.3(0.1, 2.4)	**12.3(4.5, 33.6)**	2.2(0.6, 9.1)
**Residence**	Urban					**4.4(2.5, 7.7)**	1.01(0.4, 2.5)
	Rural					1	1
**Monthly Income (in Birr)**	< 1,170	1	1	1	1	1	1
	> = 1,170	**6.4(2.02, 20.3)**	1.6(0.2, 13.8)	**2.7(1.5, 4.9)**	**5.6(2.04, 15.4)**	**4.6(2.9, 7.4)**	**2.9(1.4, 6.2)**
**Matrimonial status**	Single	1	1	1	1	NC	NC
	Married	**6.2(1.9, 20.1)**	**11.3(1.2, 110.2)**	0.49(0.2, 1.1)	1.1(0.3, 3.7)	NC	NC
	Widowed	——	——	2.2(0.5, 9.9)	1.9(0.2, 16.2)	NC	NC
	Divorced	0.2(0.04,1.5)	1(0.03, 41.8)	0.5(0.1, 1.9)	2.4(0.3, 21.5)	NC	NC
**Distance to health facility**	<10 km	NC	NC	1.6(0.9, 2.9)	**3(1.2, 8.4)**	**2.5(1.4, 4.3)**	**3(1.2, 7.3)**
	> = 10 km	NC	NC	1	1	1	1
**Diseases condition**	Acute	**4.5(1.6, 12.5)**	1.8(0.2, 14.8)	**9.2(4.9, 17.1)**	**8.9(2.4, 33.3)**	**7.5(4.6, 12.5)**	**7.2 (2.8, 18.9)**
	Chronic	1	1	1	1	1	1
**Perceived severity**	Yes	**7(2.4, 20.1)**	**6.6(1.1, 40.9)**	**3.2(1.8, 5.9)**	**2.5(1.1, 5.9)**	**4.8(2.9, 8.1)**	**3(1.6, 5.9)**
	No	1	1	1	1	1	1
**Access to health information**	Yes	NC	NC	**2.8(1.3, 6.5)**	1.4(0.4, 4.9)	**3.9(1.8, 8.5)**	1.3(0.4, 4.1)
	No	NC	NC	1	1	**1**	1

COR and AOR–Crude and Adjusted OR, NC–Not candidate: the variable was not a candidate variable for multiple logistic regression

As shown in the adjusted model, monthly income, distance to health facility, disease condition and perceived severity were found to be significant factors for seeking healthcare service use among rural households. Accordingly, rural households with monthly income above 1,170 birr were 6 times more likely to seek healthcare as compared to those who earn less than 54 USD (AOR = 5.6, 95% CI: 2.04, 15.4). Households located within 10 KM distance from health facility were 3 times (AOR, 3; 95% CI, 1.2–8.4) more than likely to seek healthcare than those located more than 10 KM. Rural households who perceived illness as acute were 9 (95% CI, 2.4, 33.3) time more likely to seek healthcare than who perceived chronic. In addition, the odds of health seeking behavior among those who perceived serious illness was 2.5 times higher (AOR, 2.5; 95%CI, 1.1, 5.9) than those who didn’t.

The third model was fitted to assess the overall factors associated with healthcare seeking behavior. Accordingly, monthly income, distance from health facility, disease condition and perceived severity of a disease were found to be significantly associated with healthcare seeking behaviour. Households with monthly income greater than 1,170 Birr were three times more likely to seek healthcare as compared to their counterparts (AOR = 2.9, 95%CI: 1.4, 6.2). Households within 10 kms distance from health facility sought healthcare three times (AOR = 3; 95% CI, 1.2–7.3) more likely than those living above 10 kms distance. The odds of healthcare seeking behavior among those who perceived disease condition to be acute were nearly seven times higher (AOR, 7.2; 95%CI, 2.8, 18.9) than those who perceived chronic disease condition and those who perceived illness as serious were three times more likely (AOR = 3, 95% CI, 1.6, 5.9) to receive healthcare compared to those who didn’t.

## Discussion

We have assessed health care seeking behavior for perceived morbidity between urban and rural households and found that three out of five households sought health care, with higher rate in urban than rural residents. This showed slight increment in both urban and rural households compared to a study conducted in North Ethiopia [26] that could be due to improvements in health extension program as well as increase in access to health facilities [27, 28]. But still special attention should be given for rural households compared to urban households as more than 80% Ethiopian population is rural resident [29].

The current study also revealed a 43% prevalence of self-medication practice. This is below the finding from Benin’s [30]. A study conducted in South Africa showed that about 50% of those who treated themselves were unsuccessful which is higher than our finding [1]. The plausible justifications could be due to the existence of less accessibility to health facilities, widespread unlicensed drug sellers and difference in illiteracy proportion [26, 31–34]. This call an action towards improving modern medicine use.

Monthly income was significantly associated with health seeking behaviors of both urban and rural households with higher monthly income associated high higher health seeking behavior. This was corroborated by studies from Georgia [35], Congo Republic [23] and Mongolia [22]. The plausible justification could be better income level association with accessibility and awareness about modern medicine [36]. The probability of seeking health care among married urban residents was higher than among single ones. This is not dissimilar with findings from studies in Jamaica[16] and Mongolia[22].

Distance from health facility was another factor for the overall health seeking behavior and this association was only for rural residents. However, when the difference was analyzed by residence, significant difference was observed among rural households. This is relatively similar with a findings from Jimma, southwest Ethiopia [37]. The existence of significant difference of health seeking behavior between rural and urban via distance is not surprising due to physical proximity of health facility deters health service utilization [36, 38]. This implies that health seeking behavior does also need addressing structural barriers.

Similarly, perceived condition of a disease was a reason for the overall health seeking behavior. However, when the difference was analyzed by residence, significant difference was observed among rural than urban households. Better health seeking behavior was recorded among those with acute disease condition than with chronic illness. This is consistent with other findings from Jamaica [16] and Vietnam [39]. The nature of acute illness which needs emergent action might let people to seek health care more. However, concern should be given as chronic illness are rising currently even in the resource meager countries [40]. Perceived severity was significantly associated with health care seeking behaviour in which households with severe illness perception were more likely to seek healthcare than non-severe illness in both residences. The plausible explanation might be due to personal fear towards the condition of diseases and its complication [41].

Worth noting limitations should have noted in this study. The study included perceived illnesses of only two months; however, there might be seasonal variation in the prevalence and incidence of disease in the study area. This might lead to over or under estimation of the proportion of healthcare seeking behavior. The possibility of social desirability bias was also high i.e. the modern health service utilization rate might be overestimated. The nature of cross-sectional study design doesn’t show temporal relationship or causality. Recall bias might also be there for some variables like age.

## Conclusions

In summary, our findings agreed with the findings of previous reports. The overall healthcare seeking behavior of households for perceived illness was satisfactory though the rate was lower in rural compared with urban households. This cues to work on accessibility and promotion of healthcare on the majority of the population of the country. Self-medication was also widely practiced in the study area that required increasing further awareness. Further consideration should also be given for the risk factors including income, matrimonial status, distance from health facility, disease condition and perceived severity of a disease. We recommend further nationwide research on the issue.

## Supporting Information

S1 File English Version Questionnaire. This is a tool used to assess the health seeking behavior of urban and rural households in southwest Ethiopia.
(DOCX)
