# Supplementary material for: Health Care Seeking Behavior in Southwest Ethiopia
Source: PLoS One. 2016 Sep 14;11(9):e0161014. doi: 10.1371/journal.pone.0161014 (PMC5023186; doi:10.1371/journal.pone.0161014)
Supplement: S1 File — This is a tool used to assess the health seeking behavior of urban and rural households in southwest Ethiopia. (DOCX) [file pone.0161014.s001.docx]

# S1 File. English Version Questionnaire. This is a tool used to assess the health seeking behavior of urban and rural households in southwest Ethiopia.

This questionnaire is prepared for collecting information on healthcare seeking behavior for perceived morbidity at household level in Esera Woreda Dawro Zone.

Consent form

001. Questionnaire identification number ______ 002. Region: SNNPR 003. Zone: Dawro 004. Place: Esera woreda 005. House number ----------

Greetings

Introduction:

My name is ______________________. I am employed as a data collector in a survey conducted by the collaboration of, Jimma university college of public health and Medical sciences, and Department of Epidemiology so as to assess associated factors for healthcare seeking behaviour, from where you got treatment, the type of sickness you have faced, the type of healthcare providers search for, decision making for healthcare seeking and the role about the traditional medicine, drugs from local shops for health seeking behavior at household level. Your name will not be written on this form and will by no means be used with any information you tell me. However, your frank responses to these inquiries will help us better realize anything people think and do about healthcare seeking behavior during sickness. I would really appreciate your aid in replying to this study. Would you be willing to take part?

1. Yes 2. No

Signature of interviewer confirming that informed consent will be give verbally by respondent. ---------------

Checked by supervisor: Name_________ Signature______________ Date______

**I Socio-Demographic Information**

| **S.No** | **Questions** | **Responses** | | | **Code** | | |
| --- | --- | --- | --- | --- | --- | --- | --- |
| 101 | household code | _____________________ | | |  | | |
| 102 | Age in years | ________________ | | |  | | |
| 103 | Sex | 1. Male___ 2. Female ______ | | |  | | |
| 104 | Residence | 1.Urban ___ 2.Rural__ | | |  | | |
| 105 | Occupation of respondents | 1. Housewife_2.Farmer__3.Government employee___ 4. Private Business__ 5.Student__ 6. Merchant_____ 7.other(specify) | | |  | | |
| 106 | Religion | 1. Orthodox__ 2. Protestant__ 3. Catholic__ 4. Muslim__ 5.Others____ | | |  | | |
| 107 | Marital status | 1. Single__2. Married__3.Widowed__ 4.Divorced___ | | |  | | |
| 108 | If married for Q107 what is your Family structure? | 1. Monogamy_____2.Polygamy | | |  | | |
| 109 | Family size | | 1. $\leq$4___ 2.>4 | |  | | |
| 110 | Ethnic group | 1. Dawro__ 2.Hadya__ 3.Wolayta__ 4.Kambata__ 5. Amhara__6.Others__ | | |  | | |
| 111 | Monthly income | 1. $<$1170 ETB__ 2.$\geq$ 1170 ETB__ | | |  | | |
| 112 | What the source of income is during illness for sought of care? | 1. Cash ____ 2. Selling kind 3. Free service | | |  | | |
| 113 | Educational status | 1. Illiterate__ 2.Primary education__ 3.Secondary education 4. Graduated from college | | |  | | |
| **II. Personal Factors** | | | | |  | | |
| 201 | Is there anyone who has any illness past 2 months in your household? | | 1. Yes 2.No | |  | | |
| 202 | What is the age of person with illness | | _____________years | |  | | |
| 203 | What is the disease condition? | | 1. acute 2.chronic | |  | | |
| 204 | If yes to q201 have you got any treatment? | | 1.no 2.yes | |  | | |
| 205 | If yes for Q203 from where have you got care? | | 1. Health Post 2 Health Centre 3 Hospital 4 Private clinics 5. Self medicated 6 Used traditional medicine | |  | | |
| 206 | If yes to q204 how many days after the onset of disease symptoms did you get care from a provider? | | 1. Immediately as illness started 2. When it goes worse 3. When it reliefs by its own | |  | | |
| 207 | If no for Q204 what is the reason? | | 1. Thought sickness is incurable 2. Symptom is not severe 3.Assumed that getting well from symptom without treatment 4. Do not know where it can be treated 5.No effective treatment is available 6.Lack of time 7 Lack of money 8. Long distance 9. Others______ | |  | | |
| 208 | If traditional for q204 from where have you got treatment? | | 1. Wegesha 2. Tsebel 3.Spiritual prayer 4. Herbalist 5.Kalicha 6.TBAs | |  | | |
| 209 | For Q203 who decided to seek treatment from Health facilities? | | 1Father 2.son 3.daughter 4.Mother 5.patient him/herself 6health professional or friends | |  | | |
| 210 | How do you perceive your general health status? | | 1.Good___ 2. Poor___ | | If Good skip to Q211 | | |
| 211 | If for Q210 your health status is poor would you seek healthcare facilities for medical consultation? | | 1. Yes___ 2. No___ | |  | | |
| 212 | If for Q203 disease is chronic do you have follow up at any health facility for consultation of medical treatment? | | 1. Yes___ 2. No___ | |  | | |
| 213 | If yes for Q211 at where do you follow? | | 1. Modern health facility 2.Traditional medicine | |  | | |
| 214 | If traditional for q212 where have you following? | | 1.Wegesha 2.Tsebel 3.Spiritual prayer 4.Herbalist 5.Kalicha 6.self medication | |  | | |
| 215 | If modern health facility for q212 where have you following? | | 1.Health post 2.Health center 3.Hospital 4.Private clinic | |  | | |
| **III. Health Institutional Variables** | | | | | | | |
| 301 | Do you think there is modern healthcare facilities access in your area? | | | 1. Yes___ 2. No___ |  | | |
| 302 | Is there Health post access? | | | 1 yes 2 no |  | | |
| 303 | Is there Health center access? | | | 1 yes 2 no |  | | |
| 304 | Is there Hospital access? | | | 1 yes 2 no |  |  |  |
| 305 | Is there Private clinic access? | | | 1 yes 2 no |  |  |  |
| 306 | For q303 health center how much far from your home? | | | 1. less than 10 km 2. more than 10 km |  | | |
| 307 | For q304 Hospital how much far from your home? | | | 1. Less than 10 km 2. more than 10 km |  |  |  |
| 308 | Does the cost of care influence your choice to health care services? | | | 1.yes  2.no |  | | |
| 309 | Do you feel healthcare staffs have good approach for you where you have utilized healthcare services? | | | 1. Yes___ 2. No___ |  | | |
| 310 | In your opinion and expectations which health care provider do you need at health care facilities? | | | 1. Doctors__ 2. Health Officers 3.Nurses___ 4.Midwives___ |  | | |
| 311 | Do you feel that you are satisfied with the behavior and accountability of health service providers? | | | 1. Yes___ 2. No___ |  | | |
| 312 | Are you satisfied with the treatment given at health facilities? | | | 1. Yes 2. No |  | | |
| 313 | In your opinion your dignity and respect is maintained as receiving care from health care providers? | | | 1. Yes 2. No |  | | |
| 314 | Have you received your needed care at the right time, when you visited to health care services? | | | 1.Yes 2.No |  | | |
| **IV. Health Belief and Related Other Variables** | | | | | | | |
| 401 | Do you tell any type of illness for health care providers? | | - - - 1. Yes       2. No | | | |  |
| 402 | If your answer is yes for Q204 what type of disease have you diagnosed there? | | 1. malaria 2.typhoid 3.gastritis 4.pneumonia 5.others (specify) 6.I don’t know | | |  | |
| 403 | How much do you value your health and try to keep it? | | 1. Very much 2.low 3.very low | | | |  |
| 404 | Do you feel shame from expression of your disease, talking about it and getting treatment for it? | | 1. Yes 2. No | | | |  |
| 405 | Do you believe from which source of care is beneficial for people who are sick? | | 1. Modern 2. Traditional 3. Both | | |  | |
| 406 | Which health facility do you trust and prefer? | | 1. Government health facilities 2. Private clinics | | |  | |
| 407 | What do you think the result of delay to seek treatment and detection? | | 1. Death 2.Delay relief time   3.Reduce treatment importance | | |  | |
| 408 | For Q204 is the illness was serious? | | 1. Yes 2. no | | |  | |
| 409 | Would you prefer a self-treatment by yourself? | | 1. Yes 2. no | | |  | |
| 410 | If yes for Q409 what is the most important reason for preferring self-treatment for the disease? | | 1. I know treating myself 2. Diseases is not serious 3.It is cost effective 4.Maintain confidentiality 5.In health care there is long waiting time 6. In health care reception is not good 7.Others_________ | | |  | |
| 411 | If your answer is choice 1 for Q409 how do you treat yourself? | | 1. Purchase drugs from shops 2. I got drugs from others 3.others(specify) | | |  | |
| 412 | If yes for Q408 do you think you treated yourself successfully? | | 1. Yes 2.No | | |  | |
| 413 | Do you have access to needed information for care receiving? | | 1.Yes 2.No | | |  | |
| 414 | If yes for Q412 from where do you often get health information? | | 1. HEWs 2. Radio 3. Television 4. Newspapers 5. Magazines 6. Internet 7.friends | | |  | |
| 415 | What is the cause for diseases | | 1 natural source 2. Man made cause like lack of sanitation 3. Others specify______ | | |  | |
